# Distribution and prediction of catalytic domains in 2-oxoglutarate dependent dioxygenases

**DOI:** 10.1186/1756-0500-5-410

**Published:** 2012-08-04

**Authors:** Siddhartha Kundu

**Affiliations:** 1 Department of Biochemistry, Army College of Medical Sciences, Delhi Cantt., New Delhi, 110010, India

**Keywords:** Hidden Markov Model, Facial triad, Ferryl, Dioxygenase

## Abstract

**Background:**

The 2-oxoglutarate dependent superfamily is a diverse group of non-haem dioxygenases, and is present in prokaryotes, eukaryotes, and archaea. The enzymes differ in substrate preference and reaction chemistry, a factor that precludes their classification by homology studies and electronic annotation schemes alone. In this work, I propose and explore the rationale of using substrates to classify structurally similar alpha-ketoglutarate dependent enzymes.

**Findings:**

Differential catalysis in phylogenetic clades of 2-OG dependent enzymes, is determined by the interactions of a subset of active-site amino acids. Identifying these with existing computational methods is challenging and not feasible for all proteins. A clustering protocol based on validated mechanisms of catalysis of known molecules, in tandem with group specific hidden markov model profiles is able to differentiate and sequester these enzymes. Access to this repository is by a web server that compares user defined unknown sequences to these pre-defined profiles and outputs a list of predicted catalytic domains. The server is free and is accessible at the following URL ( http://comp-biol.theacms.in/H2OGpred.html).

**Conclusions:**

The proposed stratification is a novel attempt at classifying and predicting 2-oxoglutarate dependent function. In addition, the server will provide researchers with a tool to compare their data to a comprehensive list of HMM profiles of catalytic domains. This work, will aid efforts by investigators to screen and characterize putative 2-OG dependent sequences. The profile database will be updated at regular intervals.

## Background

Dioxygenases, which include pterin- and 2-OG-dependent, Rieske di-hydroxylases and extradiol dioxygenases, have a conserved triad (His-Asp/Glu-His) of amino acids that are responsible for product formation [1]. The 2-OG dependent subgroup comprises members that are non-haem in character, require iron (II), and 2-oxoglutarate as a co-substrate for catalysis. Members of this superfamily are ubiquitous in nature, possess a DSBH fold (Double Stranded Beta-Helical), and the major coordinating amino acids are (HX[DE]X_n_H). Iron interacts with the pair of histidine residues and aspartate/glutamate along one face of a distorted octahedral sphere, whilst, the other face is normally coordinated by three molecular waters. In the presence of 2-oxoglutarate (bi-dentate), the last dative covalent bond is with the substrate. The accepted general route to α-KG dependent catalytic conversion, requires, an increase in the oxidation state of iron (Fe^II^ → Fe^III^-superoxo → Fe^IV^-peroxo) to a high-spin reactive ferryl intermediate (Fe^1V^ = O) [2], proton abstraction, and substrate radical formation. The transformation itself could be an oxidative- introduction of a hydroxyl group, simultaneous removal of adjacent hydrogen atoms (desaturase activity), sulfate cleavage, and cyclopentane-, stereoisomer-, chlorinated adduct- formation [3-8]. These enzymes participate in hypoxic signaling, DNA repair, stress response mechanisms, lipid and growth factor metabolism, and biodegradation of herbicides [9-15].

Existent, publically accessible computational tools and databases use homology studies to cluster proteins with 2-OG dependent function. These, provide information on sequences with evidence of common ancestry (pairwise sequence identity > 30%). Hidden Markov Models, are theoretically sound formulations of stochastic processes, being used with increasing frequency in computational biology. The output of a HMM is, a markov chain of likely consecutive states, along with their associated transitional probabilities. This class of machine learning methods is well suited to comparing divergently evolved sequences (pairwise sequence identity ~ 10-25%). InterPro is a database of protein signatures that combines information from several sources, and is used as an automatic annotation tool for new sequences. There are a number of Hidden Markov Model based predictors of protein function and classification. Pfam is a repository of protein families formed by sequence and structural similarity, and organization of distant domain architectures; SMART searches protein sequences for pre-defined regulatory domain architectures using Pfam, signal peptides, trans-membrane helices, regions of low complexity, and internal repeats; SUPERFAMILY and Gene3D integrate fold and domain data with genomic and taxonomic information to provide a comprehensive resource for proteins of interest [16-20]. These algorithms, despite providing initial pointers to the reaction chemistry of novel 2-OG dependent sequences, are unable to segregate closely related proteins with reference to their substrate preferences. Other tools focus on factors that influence intra-cellular location, propensity for protein-protein interaction, organelle targeting, and sequence patterns, rather than active-site composition and catalysis (SMART, PROSITE) [18,21].

The utilities *vide supra*, are protein sequence/structure specific. In this work, I have used a reverse look-up strategy to infer function of related proteins from the nature and similarity of the substrates catalyzed. The prediction protocol uses, in-house coded PERL scripts in conjunction with existing protein analysis tools, to create a profile database. This, is then compared with user-defined sequences, and the presence/ absence of alpha-KG dependent function and a suitable catalytic profile are suggested.

## Methods

### Computational Tools used in this work

Structural data was downloaded from the RCSB PDB server (Research Consortium for Structural Bioinformatics Protein Data Bank) [22]. Pair wise analysis of structures was done using DaliLite [23]. Analysis of active site residues was done with the SPDBV (Swiss PDB viewer), alignments and cladograms were generated with the STRAP suite of programs (Structural Alignment of Proteins), and HMMER 3.0 was used for model building, analysis, database construction, and similarity studies with user defined input sequences [24-26]. All the above software was downloaded and installed locally. Sequence ids and information on predicted domains and were from UniProtKB in association with InterPro, Pfam, SMART, and PROSITE [16-18,21,27].

**Figure 1:**
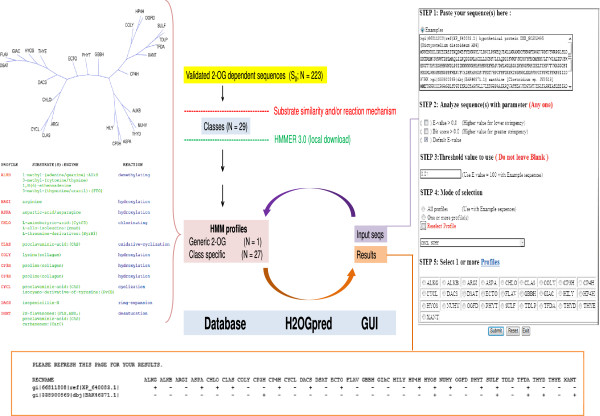
**Outline of protocol used to predict catalytic domains in user-defined sequences.** Salient features of H2OGpred: the HMM profiles used, formulation of the query required to search the database for suitable matches, and a summary of the profiles found in the sequence(s) of interest. Other details include (not shown): instructions for use, general scheme of the 2-OG dependent reaction, and detailed scores used by HMMER-3.0 for profile assignment.

I coded the PERL scripts needed to interface the front- and back- ends of the server with HMMER-3.0 and perform other miscellaneous tasks. The GUI (Graphical User Interface) for input and the results page were coded and designed by me using HTML (Hyper Text Markup Language) and CSS (Cascading Style Sheets). A concise workflow, along with salient features of H2OGpred is presented (Figure 1).

### Dataset creation and initial analysis

Several alpha-ketoglutarate enzymes have associated empirical data present in medical literature. This constitutes: demonstration of activity *in vitro* and *in vivo* (EC 1.14.11.x), transcript data with biochemical and/or physiological function, and the presence of a structure. These sequences (N = 223), were collated and comprised the template set (S_0_; Additional file 1: Table S1). This was divided into a training- (S_1T_, N = 81) and a validation- (S_1V_, N = 142) set of sequences. Early work to assess the catalytic profile of each member of S_0_, was done by searching for suitable domains in publically available databases (Additional file 2: Table S2). The feasibility of a substrate centric classification of αKG-dependent enzyme members was investigated subsequently. This was done by analyzing proteins with considerable structural similarity (Z score ≥ 20.0, Additional file 3: Table S3), and in complex with dissimilar preferred substrates and/or analogs. Differences in the amino acids that lined the substrate pocket were tabulated.

### Construction of profile database and server

The 2-OG dependent enzymes are multi-functional catalysts. Clavaminate synthase (EC 1.14.11.21) transforms proclavaminate and/or analogs by introducing a hydroxyl group, double bond, and effecting a ring closure reaction [3]. The 2 S-flavanones, are similarly desaturated and hydroxylated by flavone-, flavonol-, and anthocyanidin- synthases (EC 1.14.11.x, x = 19, 22, 23) and flavanone 3-dioxygenase (EC 1.14.11. 9) [4]. Integrating prior information for each of the above enzymes (S_0_), such as reaction chemistry, participating macromolecules, simple organic compounds which include endogenous (amino acids, acyl-CoA molecules) and exogenous (herbicides, pesticides, detergents), and molecular and atomic level detail (transferred element or functional group), a secondary filter was set up. The resultant sub-clusters constituted overlapping members, were descriptively annotated, profiled as HMMs, and a sequence signature pattern composed of alignment specific identical amino acids, was assigned to each (Additional file 4: Table S4). In addition, class specific consensus sequences were generated and aligned. This data was used to create an unrooted cladogram (Figure 1).

The complete list of HMMs (N = 28), comprised, a superfamily (S_1T_) and group (S_2_; by analogy) specific models. The selection of sequences for the generic, αKG-profile (ALKG) was done to ensure adequate coverage and even sampling of S_0_. Classes with single enzyme members were excluded (ATSK; PTLH). The profile database created is available as (Additional file 5: Table S5; aKG-profile-database.hmm). Interface to this repository is through H2OGpred, a server that accepts user defined protein sequences, and predict domains specific to a particular substrate.

## Findings

**Figure 2:**
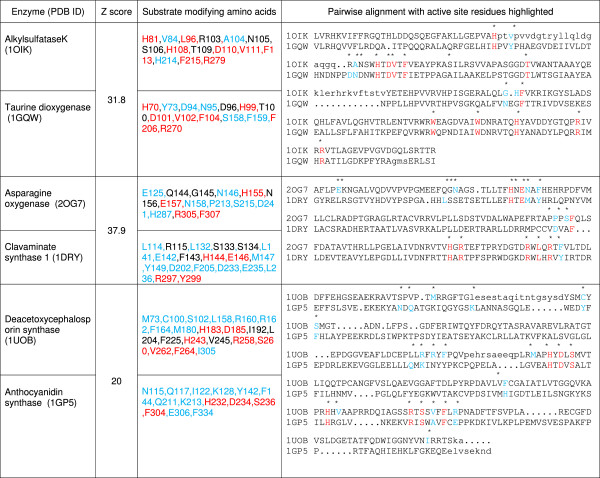
**Alignment and active site analysis of structurally similar pairs of proteins.** Inter-molecular substrate modifying residues (1 or more atoms within 5 A^0^ of atom(s) of compound of interest) have been tabulated and compared. Color scheme for highlighting: Red – identity, Blue- protein specific, Black – amino acids with their side chains pointing away from the substrate, suggesting a structural role.

This study highlights and discusses the following characteristics of the 2-OG dependent superfamily. There are observable differences in the reaction mechanisms and/or substrates transformed in structurally related enzymes (Table 1, Figure 2). These variations are with reference to the amino acids that border the substrate binding pocket, interact with 2-OG, Fe(II), and participate in alpha-KG specific domain formation. A detailed analysis of predicted domains in previously collated sequences (S_0_, Additional file 2: Table S2), using publically accessible tools, revealed that, the TauD family (PF02668, sequences = 4205, non-redundant PDB ids = 8), consists of enzymes such as: taurine dioxygenase, alkylsulfataseK, asparagine oxygenase, carbapenem synthase C, L-arginine-beta-hydroxylase, and gamma-butyrobetaine hydroxylase among others. Similarly, the PhyH family (PF05721, sequences = 2319, non-redundant PDB ids = 3) encompasses activities of phytanoyl-CoA-dioxygenase, ectoine hydroxylase, and pentalenolactone synthase. Interestingly, all the above catalyze different substrates, clearly demonstrating the lack of discriminatory indices in current literature to delineate function in similar proteins.

**Table 1:** Comparison between structurally similar 2-OG dependent proteins

	**Taurine dioxygenase (TauD)**	**Alkylsulfatase (AtsK)**
**Organism**	*Escherichia coli*	*Pseudomonas putida*
**alpha/beta content**		
DSBH (core)	β (5–8,14-17)	β (1–7;13–16)
Extended	β (9–10), α (1–6)	β (8–12), α (3–5)
**PDB id**	1GQW (Ref. [229])	1OIK (Ref. [227])
**Sequence identity (%)**	41-42	
**Z score (rmsd)**	31.8-34.4 (1.2-1.3)	
**Active site geometry (amino acid nos.)**		
Sphere of radius 5 A^0^ (Fe/2-OG/Substrate)	18	17
Identical residues	13	
**Reaction catalyzed**	sulfate cleaving activity	
**Substrate profile**	taurine (sulfonic acids)	aliphatic sulfate esters
	**Phytanoyl -CoA hydroxylase(PAHX)**	**Pentalenolactone hydrolase(PtlH)**
**Organism**	*Homo sapiens*	*Streptomyces avermitilis*
**alpha/beta content**		
DSBH (core)	β (6,8-13) β (2-4, 7-10)	
Extended	β (1–2,5,15) β (1), a (1-6)	
**PDB id**	2A1X (Ref. [220])	2RDN (Ref. [226])
**Sequence identity (%)**	20	
**Z score (rmsd)**	17.1 (2.9)	
**Amino acids within sphere of radius 5 A**^**0**^**(Fe/2-OG/Substrate)**	12	18
**Reaction catalyzed**	hydroxylation	ring closure
**Substrate profile**	phytanoyl CoA (medium chain)	1-deoxypentalenic acid

As an alternate approach to this problem, I, hypothesized that substrate interacting amino acids in the active site might be used to further classify structurally similar enzymes. To test this rationale, select pairs of the 2-OG dependent superfamily were analyzed and compared. The results indicate, that despite similarities in the composition of the active site, subtle differences exist in the nature of these additional substrate-modifying residues (Figure 2), which, in turn could correlate to differential catalytic behavior. The sub-classes formed by utilizing substrates as clustering parameters are evolutionarily diverse (Table 2, Figure 1). Nevertheless, the frequency of identical amino acids (iaa) in the signature patterns of the profiles (iaa = 0, N = 4; iaa = 1 – 2, N = 3; iaa > 2, N = 20), suggests, the existence of a conserved domain architecture for each group (Additional file 4: Table S4). These functional profiles compare favorably with classically annotated protein specific domains using as criteria: prediction of function, redundancy, and segregation of structurally related proteins (Table 3). A catalytic domain, in this work is defined, hereafter, as a HMM of a group of sequences with similar substrate preferences and/ or reaction chemistry.

**Table 2:** Classification of 2-OG dependent dioxygenases

	Family (This work)*
**REACTION**	
Demethylation	ALKB, COLY
Chlorinating	CHLO
Ring closure (cyclization)	CYCL
Desaturation	CLAS, CYCL, FLAV
Sulfate cleavage	TDLP
Ring expansion	DACS
Ether bond cleavage	TFDA
Hydroxylation	NUHY(THYD,THYE,XANT), CP3H,CP4H,HP4H,ARGI,ASPA,ECTO,PHYT,GBBH,HYOS, PTLH
**SUBSTRATE**	
**Amino acid/protein/derivative**	
Arginine	ARGI
Aspartyl; Asparagine	ASPA
Lysine	COLY, HILY
Proline	CP3H,CP4H,HP4H
Sulfonic acids (taurine), isethionate, taurocholate	SULF (TDLP)
gamma-butyrobetaine	GBBH
Collagen	CP3H,CP4H,COLY
**Nucleotide/ nucleoside**	
Thymidine	THYD
Thymine	THYE
Xanthine	XANT
**Misc. organic**	
Pro-clavaminate	CLAS
Deacetoxycephalosporin	DACS
Ectoine	ECTO
2 S-flavanones	FLAV
Gibberellins	GIAC
Hyoscyamine	HYOS
Phytanoyl-CoA	PHYT
PAA based pesticides/ herbicides	TFDA
Cyclopentane	PTLH
Aliphatic sulfate esters	ATSK
Translation in eukaryotes (eIF2α)	OGFD

(*) Note: Details of protein sequences used in this analysis (uniprot & PDB ids, references) are included as Additional file1**: Table S1.**

**Table 3:** Comparative analysis of catalytic domains of template sequences *

	**InterPro**	**PFAM**	**SMART**	**PROSITE**
**Group 1**				
**FLAV**	Oxoglutarate/Fe-dep_oxygenase	2OG-FeII_Oxy		FE2OG_OXY
	Isopenicillin-N_synthase			
**GIAC**	Oxoglutarate/Fe-dep_oxygenase	2OG-FeII_Oxy		FE2OG_OXY
	Isopenicillin-N_synthase			
**HYOS**	Oxoglutarate/Fe-dep_oxygenase	2OG-FeII_Oxy		FE2OG_OXY
	Isopenicillin-N_synthase			
**DACS**	Isopenicillin-N_synth_CS	2OG-FeII_Oxy		FE2OG_OXY
	Oxoglutarate/Fe-dep_oxygenase			IPNS_1
				IPNS_2
**THYE**	Oxoglutarate/Fe-dep_oxygenase	2OG-FeII_Oxy		FE2OG_OXY
**Group 2**				
**TDLP**	Taurine_dOase	TauD		
**ATSK**	Taurine_dOase	TauD		
**TFDA**	Taurine_dOase	TauD		
**XANT**	Taurine_dOase	TauD		
**Group 3**				
**PHYT**	Phytyl_CoA_dOase	PhyH		
**PTLH**	Phytyl_CoA_dOase	PhyH		
**CP4H**	Oxoglutarate/Fe-dep_oxygenase	2-OG-FeII_Oxy	P4Hc	FE2OG_OXY
	Pro_4_hyd_alph	P4Ha_N	ShKT	TPR
	Pro_4_hyd_alph_N	ShK		TPR_REGION
	ShK_toxin			
	TPR-contain			
	TPR-like_helical			
	TPR_repeat			
**HP4H**	Oxoglutarate/Fe-dep_oxygenase	2OG-FeII_Oxy	P4Hc	FE2OG_OXY
	Pro_4_hyd_alph	Cupin_4		ZF_MYND_1
	Cupin_JmjC	zf-MYND		ZF_MYND_2
	Znf_MYND	Ofd1_CTDD		
	Oxoglutarate/Fe-dep_Oase_C			

(*) Note: Complete domain analysis of template dataset is included as Additional file 2**: Table S2.**

## Discussion

A fundamental detail of alpha-ketoglutarate dependent catalysis is the range of substrates transformed, and the distinct reaction mechanisms deployed. This remarkable feature is, despite the presence of several common structural features such as the presence of the jellyroll fold, active site composition, and the presence of the facial-triad of residues (Table 1). Several attempts to classify these enzymes have been made previously [28,29]. Both, sequence-based studies: location of the facial triad (central; C-terminal, flavanol synthase), number of amino acids between His-X-[Asp/Glu] and the terminal His (125 a. a, taurine dioxygenase, alkylsulfatase; 57 a. a, anthocyanidin synthase; 85 a. a, phytanoyl-CoA- hydroxylase), and reaction specific structural features such as the presence, location, composition, and conformational arrangements of conserved active site residues. These approaches, albeit informative are unable to account for the catalytic spectrum observed within sub-groups of the superfamily. This is attributed to subtle modifications in the distribution patterns of a few amino acids, and may, constitute an extended active site. Thus, the presence of small hydrophobic residues in alkylsulfatase K (V84, A104), as opposed to the corresponding charged residues in the related enzymes, i.e., taurine dioxygenase (Y73, D94, N95), ensure that sulfonic acids, modified amino and bile acids, are preferred over aliphatic sulfur esters (pair 1, Figure 2). Clavaminic acid synthase 1 and asparagine oxygenase share remarkable structural similarity. The presence of a glutamic acid residue (HEH, facial triad), and high Z score, notwithstanding, CAS1 is a tri-functional catalyst with a completely different set of preferred compounds (pair 2, Figure 2). Similarly, use of 2 S-flavanones (FLAV profile) in preference to isopenicillin N (deacetoxycephalosporin synthase, EC 1.14.11.26) is a function of a few specialized residues (pair 3, Figure 2). Characterizing these residues by homology alignments and subsequent mutagenesis experiments are currently the only known means to ascribe function.

2 – OG dependent enzymes catalyze the hydroxylation of their substrates. However, this step may also occur concomitantly with other reactions. In these cases, an intermediate substrate radical is the precursor for a subsequent catalytic event. Clearly, the notion of a substrate molecule as a passive transformant is *passé*, with increasing evidence of its role in modulating catalysis. Prediction, by existing tools, of product forming domains in an enzyme specific to a particular substrate is generic, with no information on substrate specificity for a number of families (Group 1, Table 3), whilst, the same catalytic domain for a reference sequence is assigned to a number of other enzymes (Groups −2 and 3, Table 3). However, by integrating the profiles it is possible to infer the function of an unknown protein. The HP4H (hypoxia inducible prolyl 4-hydroxylase, EC 1.14.11.29) domain in a protein, is an important indicator of a role in regulating downstream genes in response to hypoxic conditions, the same may be inferred from the ensemble of predicted domains (Pro_4_hyd_alph; Znf_MYND; Cupin_JmjC). Similarly, CP4H (collagen prolyl 4-hydroxylase, EC 1.14.11.2) function may be postulated by integrating its domain profile (Pro_4_hyd_alph; TPR_helical; TPR-contain).

These latter examples (Group 4, Table 3) suggest that absence of prior information will limit the utility of this substrate centric, profile assignment process. A sequence with no suitable profile matches might require a comparison by homology studies to existing/ computationally annotated protein sequences. Despite these constraints, this novel schema is able to categorize closely related protein sequences. As biochemical details of a greater number of enzymes emerge, it will be possible to develop improved docking algorithms and statistical models of the chemical signature of a substrate molecule. This could then predict active site conformers of a particular enzyme for an individual substrate.

### Description of H2OGpred

The web server works by comparing sequences with each of these pre-defined HMM profiles (Figure 1). There is a brief introduction to the salient features of α-KG dependent enzymes, and general instructions of use. Users can paste their sequences of interest, select a threshold parameter and value and search the profile database. Output files comprise a tabular summary of suitably matched profiles, and detailed statistics with pair wise alignments. Details of the profiles are present as a hyperlink and combined with the result as a separate file. New users may utilize the examples option to analyze and view preliminary results. The server has been tested with approximately 250 sequences, pasted at once.

### Validation of H2OGpred as a predictor 2-OG dependent catalysis

To verify functionality of the server, proteins that were not used to construct the generic profile (S_1V_), were analyzed further. The server was correctly able to predict the presence of a single 2-OG domain in all test sequences (N = 142). Further, two novel sequences, have been experimentally validated (unpublished data) with demonstration of catalytic activity towards their preferred substrates in concurrence with the top scoring profiles assigned by the server.

## Conclusions

The 2-oxoglutarate dependent enzymes are amongst the largest group of non-haem dioxygenases, rivaling the more established mediators of xenobiotic metabolism, the cytochrome P450 family of haem monooxygenases. Current information on novel non-haem 2-OG dependent iron (II) enzymes is sparse, and relies on sequence/structure-based homology studies. In addition, complete biochemical characterization often necessitates prior knowledge of potential substrates. Here, I, have compiled a list of enzymes previously validated by several workers [30-236], and categorized them on the similarity of the reactions they catalyze, and/or, of the compounds they modify. The resulting HMMs are then used to construct a map of putative catalytic domains, thereby suggesting, a list of potential molecules that new, uncharacterized sequences might transform.

Enzyme members of the αKG-dependent superfamily are downstream mediators of a stimulus-induced-compensatory stress response in several organisms. This includes cycles of, cellular hypoxia and altered expression patterns of regulatory and effector genes, exposure to herbicides and arsenic with activation of catabolic pathways, and high salinity and thermal stress with overproduction of compatible solutes. Thus, an insight into the reaction chemistry of these proteins has the potential to aid development of newer classes of antimicrobials, bio-degradable compounds, and efficacious metabolic regulators.

### Availability

Project URL: http://comp-biol.theacms.in/H2OGpred.html.

Usage: Free and no login required.

## Abbreviations

2-OG, 2-oxoglutarate; HMM, Hidden Markov model; GUI, Graphical user interface.

## Competing interests

The authors declare that they have no competing interests.

## Author’s contribution

SK manually collated all the sequences and their references, carried out the computational analysis, constructed the server, designed the GUI, wrote all the code, and the manuscript.

## Supplementary Material

Additional file 1 **Table S1.** Uniprot and PDB ids of sequences used in this work. Click here for file

Additional file 2 **Table S2.** Comparative domain analysis of template sequences. Click here for file

Additional file 3 **Table S3.** Pair wise structural alignment of selected proteins. Click here for file

Additional file 4 **Table S4.** Analysis of HMM profiles with highlighted active site residues. (PDF 29 kb) Click here for file

Additional file 5 **Table S5.** Link to HMM profile database “profile-DB\aKG-profile-database.hmm”. Click here for file
