# Supplementary material for: Distribution and prediction of catalytic domains in 2-oxoglutarate dependent dioxygenases
Source: BMC Res Notes. 2012 Aug 4;5:410. doi: 10.1186/1756-0500-5-410 (PMC3475032; doi:10.1186/1756-0500-5-410)
Supplement: Additional file 1 — Table S1. Uniprot and PDB ids of sequences used in this work. [file 1756-0500-5-410-S1.pdf]

Table S1: uniprot ids of template sequences used in this work

Prot. Family (This study)	Uniprot id	PDB id	Refs.
ALKB	**P05050	2FD8	30
(30 -35)	**Q6NS38	2IUW	30
	**Q96Q83	3BUC	31
	**Q9C0B1	3LFM	32
	Q5XKL0		33
ARGI	**Q05581	1DRT	36
(36 - 39)	**Q6WZB0	2WBP	37
	Q05582		38
ASPA	Q9NWT6	1H2L	40
(40 - 48)	Q9Z4Z5	2OG7	41
	Q12797	3RCQ	42
	*Q28056		43
	*Q8BSY0		44
	Q5HZW3		45, 46
	*Q9GQ82		44
ATSK	**Q9WWU5	1OIK	227, 228
(227, 228)			
CHLO	**Q9RBY6	2FCT	49
(49 - 52)	**D0VX22	3GJB	50
	*Q87W55		51
CLAS	**Q05581	1DRT	36
(36, 38)	*Q05582		38
COLY	Q6NYC1	3K2O	53
(53 - 63)	*O00469		54
	*Q63321		55
	*Q5U367		56
	Q9R0E1		57
	Q9R0B9		57
	Q9R0E2		57
	Q20679		58
	Q91ZW6		59
	*O60568		60
	Q811A3		56
	A8WIN7		61
	A4U7F8		61
	Q1L618		62
CP3H	O09345	1E5R	64
(64 - 72)	*Q4KLM6		65, 66

(*) sequence id used to create generic 2-OG profile

(**) PDB id of sequence used to create generic 2-OG profile

	*Q9R1J8		67				
	*Q8IVL6		68				
	*Q8IVL5		68				
	Q32P28		69				
	Q3V1T4		69				
	Q8CG70		68				
	Q6JHU8		70				
	Q8CG71		68, 71				
CP4H	**A8J7D3	2JIG	73				
(73 - 82)	**Q81LZ8	3ITQ	74				
	P13674	2V5F	75				
	O15460		76, 77				
	Q05DA4		76, 77				
	*Q9ZW86		78				
	*F4JAU3		79				
	Q84406		80				
	Q75ZI4		81				
CYCL	Q05581	1DRT	36				
(36, 38, 83)	**Q9I1L4	3EAT	83				
	Q05582		38				
DACS	**P18548	1UOB	84				
(84 - 89)	*Q03047		85				
	*P11935		86				
	*P42220		87				
	P42219		88				
DSAT	Q05581	1DRT	36				
(36, 38, 90 - 105)	**Q96323	1GP5	90				
	Q9XB59	1NX8	91				
	Q7XZQ8		92				
	Q7XZQ6		93				
	Q9ZWQ9		94				
	Q96330		95, 96				
	Q05964		97				
	Q07512		98				
	Q52T40		99				
	A0EKE7		99				
	B6A7R4		100				
	D0UZK7		101				
	Q0EDG6		102				
	Q2PGC5		103				
	Q2PGC6		103				
	Q2PHJ7		103				
	Q2PHK4		103				
	Q2PHK5		103				
	B2M0X8		104				
	Q05582		38				

ECTO	**Q2TDY4	3EMR	106				
(106 - 109)	*Q6QUY7		107				
	*Q93RV9		108				
	*Q1RPP0		109				
FLAV	**Q96323	1GP5	90				
(90, 92 - 104)	Q7XZQ8		92				
	*Q7XZQ6		93				
	*Q9ZWQ9		94				
	*Q96330		95, 96				
	Q05964		97				
	Q07512		98				
	Q52T40		99				
	A0EKE7		99				
	B6A7R4		100				
	D0UZK7		101				
	Q0EDG6		102				
	Q2PGC5		103				
	Q2PGC6		103				
	Q2PHJ7		103				
	Q2PHK4		103				
	Q2PHK5		103				
	B2M0X8		104				
GBBH	**O75936	3N6W	110				
(110 - 114)	*P80193		111				
	*Q924Y0		112				
	*A6QQL2		113				
GIAC	Q39103		115				
(115 - 150)	Q0JH50		116,117				
	Q39110		118,119				
	*Q8LEA2		120				
	Q39112		119				
	Q9XFR9		120				
	Q9C6I4		121				
	O49561		121				
	Q9SQ80		122,123				
	Q9ZT84		124				
	Q39111		119				
	O64692		120				
	Q9C971		123				
	P93771		125				
	P0C5H5		116,117				
	Q9SVS8		126				
	Q9XHM5		122				
	Q3I411		127				
	O04705		127				
	Q9XG83		120				

	Q3I410		127				
	Q3I409		127				
	O04707		127				
	O04706		127				
	Q941N1		128				
	Q941N2		128				
	A7LCJ5		129				
	Q9FS86		130				
	Q9FS85		130				
	C0LZW8		128				
	C0LZW9		128				
	Q9FS84		130				
	O23762		131				
	Q8GT56		132				
	Q9M4A0		133				
	Q8GUE8		132				
	O24417		134				
	Q7X9N5		132				
	O23763		135				
	*Q8GT57		132				
	Q9M4P2		132				
	Q9M499		133				
	Q41366		136				
	*Q8GSN6		137				
	Q5D0G3		138				
	Q8GSN7		137				
	Q8GSN5		137				
	O04281		139				
	*O04280		139				
	O04282		139				
	Q4W8C0		140				
	Q4W8C3		140				
	Q4W8C4		140				
	Q4W8C1		140				
	B2MWW5		141				
	A4GVL8		141				
	A4GVL7		141				
	A4GVL9		141				
	A4GVL5		141				
	Q9ZPP4		141,143				
	Q9ZPP2		141				
	Q9ZPP3		141				
	A3FAV1		142				
	A0FK57		144				
	A7LNI6		143				
	A4GVL6		141				
	Q9ZWR7		145				
	Q9ZWR6		145				
	Q4W8C2		140				
	E1AXG4		146				

	Q96498		147				
	*E1AXG6		146				
	E1AXG5		146				
	Q9ZWQ1		148				
	Q9FXV7		149				
	Q9FXW1		150				
	Q9FXV6		148,149				
	Q9ZWP9		148				
	Q9FXW0		150				
	Q9ZWQ2		148				
	O04277		139				
HILY	**Q80Y84	2EQY	151,152				
(151 - 190)	Q9H3R0	2XML	153,154				
	O15054	2XXZ	155,156				
	Q9BY66	2YQE	157,158				
	Q6B0I6	3DXT	153,159				
	P29375	3GL6	160-162				
	Q9UPP1	3K3O	163,164				
	Q6ZMT4	3KV6	163,165				
	Q9VMJ7	2LM1	166-168				
	*Q9V333		169				
	*Q9V6L0		170				
	O15550		171				
	O70546		172				
	Q5NCY0		171,173				
	Q8N371		174				
	Q6PCM1		175,176				
	Q69ZK6		177				
	Q6ZPY7		178				
	P41229	2JRZ	179,180				
	O75164	2Q8C	181,182				
	Q09519		183,184				
	*Q9VL07		185,186				
	Q9LT40		187				
HP4H	*Q96KS0		191-194				
(191 - 204)	*Q9H6Z9		192,194				
	Q86KR9		195				
	Q9NWT6	1H2L	196-198				
	**Q9GZT9	2G19	192,194,199				
	**P40032	3KT1	200				
	Q8N543		201				
	Q11120		202				
HYOS	*P24397		205				
(205 - 211)	*O04847		206,207				
	*B2CNC8		208				
	*Q6V0J1		209				
	Q6EZB3		210				

	A9Q1G4		211				
NUHY	*Q4QZZ9		212,213				
(212 - 219)	*Q7SEI7		213				
	Q6CHN2		213				
	*B6EU02		214				
	*P86938		215,216				
	*C5I496		217				
	*Q7RYZ9		218,219				
OGFD	*Q8N543		201				
(201, 202)	*Q11120		202				
PHYT	**O14832	2A1X	220				
(220 - 225)	**Q5SRE7	3OBZ	221				
	*P57093		222				
	*O35386		223				
	*Q9ZVF6		224				
PTLH	Q82IZ1	2RDN	226				
(226)							
SULF	**Q9WWU5	1OIK	227,228				
(227 - 231)	**P37610	1GQW	229,230				
	*Q12358		231				
TDLP	P37610	1GQW	229,230				
(229 - 231)	Q12358		231				
TFDA	*P10088		232,233				
(232 - 236)	Q8KSC8		234				
	*Q700X4		234				
	*Q208A9		234,235				
	Q67FR0		236				
	*Q67FS3		236				
THYD	B6EU02		214				
(214 - 216)	P86938		215,216				
THYE	C5I496		217				
(217 - 219)	Q7RYZ9		218,219				
XANT	Q4QZZ9		212,213				
(212, 213)	Q7SEI7		213				
	Q6CHN2		213				
