# Supplementary material for: Distribution and prediction of catalytic domains in 2-oxoglutarate dependent dioxygenases
Source: BMC Res Notes. 2012 Aug 4;5:410. doi: 10.1186/1756-0500-5-410 (PMC3475032; doi:10.1186/1756-0500-5-410)
Supplement: Additional file 2 — Table S2. Comparative domain analysis of template sequences. [file 1756-0500-5-410-S2.pdf]

Table S2: Domain analysis of template sequences and comparsion with existent databases					
**This study**	**Seq. Nos.**	**InterPro**	**PFAM**	**SMART**	**PROSITE**
**ALKB**	5	Alkb.	2OG-FeII_Oxy		FE2OG_OXY
		Oxoglutarate/Fe-dep_oxygenase	FTO_CTD.		
		FTO_C.	FTO_NTD.		
		FTO_cat_dom.			
**ARGI**	3	Arginine_beta-hydroxylase.	TauD		
		Clavaminate_syn.			
		Taurine_dOase.			
**ASPA**	7	Cupin_JmjC.	Cupin_4	JmjC	JMJC
		TF_JmjC_AAH	TauD		TPR
		Clavaminate_syn.	Asp-B-Hydro_N.		TPR_REGION
		Taurine_dOase.	Asp_Arg_Hydrox.		
		Asp-B-hydro/Triadin_dom.			
		Asp_Arg_b-Hydrxlase.			
		TPR-contain.			
		TPR-like_helical.			
		TPR_repeat.			
**ATSK**	1	Taurine_dOase.	TauD		
**CHLO**	3	Chlorin_enz.	PhyH		
		Phytyl_CoA_dOase.			
**CLAS**	2	Clavaminate_syn.	TauD		
		Taurine_dOase.			
**COLY**	14	TF_JmjC.	JmjC	JmjC	JMJC
		TF_JmjC_AAH.	2-OG-FeII_Oxy	P4Hc	FE2OG_OXY
		Oxoglutarate/Fe-dep_oxygenase.	DUF971.		LYS_HYDROXYLASE
		Pro_4_hyd_alph.	TauD.		
		Procol_lys_dOase.			
		DUF971.			
		Taurine_dOase.			
		Trimethyllysine_dOase.			
**CP3H**	10	Asp_Arg_b-Hydrxlase.	2-OG-FeII_Oxy	P4Hc	ER_TARGET
		Pro_3_hydrox_C.	Asp_Arg_Hydrox.		FE2OG_OXY
		Oxoglutarate/Fe-dep_oxygenase.	Pro_3_hydrox_C.		TPR (False Negative)
		Pro_4_hyd_alph.			TPR_REGION (False Negative)
		TPR-like_helical.			

**CP4H**	9	Oxoglutarate/Fe-dep_oxygenase	2-OG-FeII_Oxy	P4Hc	FE2OG_OXY
		Pro_4_hyd_alph	P4Ha_N	ShKT	TPR
		Pro_4_hyd_alph_N	ShK		TPR_REGION
		ShK_toxin			
		TPR-contain			
		TPR-like_helical			
		TPR_repeat			
**CYCL**	3	Taurine_dOase.	TauD		
		Clavaminate_syn.			
**DACS**	5	Isopenicillin-N_synth_CS.	2OG-FeII_Oxy		FE2OG_OXY.
		Oxoglutarate/Fe-dep_oxygenase.			IPNS_1.
					IPNS_2.
**DSAT**	21	Taurine_dOase.	TauD		FE2OG_OXY.
		Clavaminate_syn.	2OG-FeII_Oxy		
		Oxoglutarate/Fe-dep_oxygenase.			
		Isopenicillin-N_synthase.			
**ECTO**	4	Ectoine_ThpD.	PhyH		
		Phytyl_CoA_dOase.			
**FLAV**	18	Oxoglutarate/Fe-dep_oxygenase.	2OG-FeII_Oxy		FE2OG_OXY.
		Isopenicillin-N_synthase.			
**GBBH**	4	2-oxoglut_dOase.	DUF971.		
		DUF971.	TauD.		
		Taurine_dOase.			
**GIAC**	81	Oxoglutarate/Fe-dep_oxygenase.	2OG-FeII_Oxy		FE2OG_OXY.
		Isopenicillin-N_synthase.			
**HILY**	23	ARID/BRIGHT_DNA-bd.	ARID.	BRIGHT.	ARID.
		Lys_sp_deMease_like_dom.	JmjC.	JmjC.	JMJC.
		TF_JmjC.	JmjN.	JmjN.	JMJN.
		TF_JmjC_AAH.	PHD.	PHD.	ZF_PHD_1.
		TF_JmjN.	PLU-1.	TUDOR	ZF_PHD_2.
		Zinc_finger_PHD-type_CS.	zf-C5HC2.		TUDOR False Negative
		Znf_C5HC2.	TPR_1		
		Znf_FYVE_PHD.	Cupin_4		
		Znf_PHD.			
		Znf_PHD-finger.			
		Znf_RING/FYVE/PHD			
		Tudor.			
		TPR-like_helical.			
		TPR-1			
		Cupin_JmjC.			

**HP4H**	8	Oxoglutarate/Fe-dep_oxygenase.	2OG-FeII_Oxy	P4Hc	FE2OG_OXY.
		Pro_4_hyd_alph.	Cupin_4		ZF_MYND_1.
		Cupin_JmjC.	zf-MYND		ZF_MYND_2.
		Znf_MYND.	Ofd1_CTDD.		
		Oxoglutarate/Fe-dep_Oase_C			
**HYOS**	6	Oxoglutarate/Fe-dep_oxygenase.	2OG-FeII_Oxy		FE2OG_OXY.
		Isopenicillin-N_synthase.			
**NUHY**	7	Taurine_dOase	TauD	DEXDc.	HELICASE_ATP_BIND_1.
		DEAD-like_helicase.	Helicase_C.	HELICc.	HELICASE_CTER.
		Helicase_C.	SNF2_N.		
		SNF2_N.			
		Oxoglutarate/Fe-dep_oxygenase.	2OG-FeII_Oxy		FE2OG_OXY.
		Isopenicillin-N_synthase.			
**OGFD**	2	Oxoglutarate/Fe-dep_Oase_C	Ofd1_CTDD.	P4Hc	FE2OG_OXY
		Oxoglutarate/Fe-dep_oxygenase			
		Pro_4_hyd_alph			
**PHYT**	5	Phytyl_CoA_dOase	PhyH		
**PTLH**	1	Phytyl_CoA_dOase	PhyH		
**SULF**	3	Taurine_dOase	TauD		
**TDLP**	2	Taurine_dOase	TauD		
**TFDA**	6	Taurine_dOase	TauD		
**THYD**	2	DEAD-like_helicase.	Helicase_C.	DEXDc.	HELICASE_ATP_BIND_1.
		Helicase_C.	SNF2_N.		
		SNF2_N.			
**THYE**	2	Oxoglutarate/Fe-dep_oxygenase.	2OG-FeII_Oxy		FE2OG_OXY.
**XANT**	3	Taurine_dOase	TauD		
