# Supplementary material for: Distribution and prediction of catalytic domains in 2-oxoglutarate dependent dioxygenases
Source: BMC Res Notes. 2012 Aug 4;5:410. doi: 10.1186/1756-0500-5-410 (PMC3475032; doi:10.1186/1756-0500-5-410)
Supplement: Additional file 3 — Table S3. Pair wise structural alignment of selected proteins. [file 1756-0500-5-410-S3.pdf]

Table S3: DaliLite analysis of selected 2-OG dependent enzyme structures (Ref. 23)						
**Ref. prot**		**Z score**	**Aligned amino acids**	**RMSD**	**Seq. identity (%)**	
**TauD**						
	**AtsK**	**31.8-34.4**	**228-242**	**1.2-1.3**	**41-42**	
	CarC	21.5-22.1	225-232	3.0-3.2	18-19	
	AsnO	18.0-18.1	224	2.9	18	
	CAS1	17.8-17.9	221-224	2.9	15	
	DAOCS	5.7	128-130	3.8-3.9	8.0-9.0	
	IPNS	5.4	134	3.6	11	
	ANS	5.1-5.4	132-135	3.8-3.9	18	
	FIH	7.3	151-152	3.9	11	
	PAHX	7.3	136	3.4	9	
	AlkBH3	6.0-6.1	119	3.4	8	
	AlkB	6	110-111	2.9	14-15	
	Ptlh	7.7-7.8	148-150	3.3-3.4	9	
	Jmjd2A	6.6-7.0	142-158	3.1-4.4	8	
	hjHDM1	7.2-7.4	139-141	3.2-3.3	7.0-8.0	
**AsnO**						
	AtsK	16.3-17.4	201-210	2.7	18	
	TauD	18.0-18.1	224	2.9	18	
	**CAS1**	**37.9**	**300**	**2.1**	**32**	
	CarC	18.7-19.2	213-218	2.7-2.8	17	
	DAOCS	6.5	152	3.8	11	
	IPNS	6.2-6.3	154-158	3.9	11	
	ANS	5.2	149	4.1	14	
	FIH	8	167	4.3	4	
	AlkBH3	6.7	144	3.8	10	
	AlkB	6.8	135	3.8	11	
	PAHX	8.5	166	3.6	8	
	Ptlh	8.4	176	3.9	13	
	Jmjd2A	7.7-7.8	178-179	5	7	
	hjHDM1	7.3	158	3.8	9	
**DAOCS**						
	Atsk	5.6-6.1	113-132	3.7-4.8	7.0-8.0	
	TauD	5.7	128-130	3.8-3.9	8.0-9.0	
	AsnO	6.5	152	3.8	11	
	CarC	5.4-5.6	118-125	3.2-3.9	8.0-9.0	
	CAS1	5.8	143	4.3	9	
	**ANS**	**21.7**	**239**	**2.9**	**20**	
	FIH	7.9	174	3.9	11	
	AlkBH3	7.6	140	3.2	9	
	AlkB	7.9	133	3.6	12	
	Ptlh	9.4	161	3.5	12	
	PAHX	10.3	169	4.3	11	
	Jmjd2A	5.3-5.4	148-153	4.1-4.7	9	
	hjHDM1	6.5	160	4	7	
