# Supplementary material for: Distribution and prediction of catalytic domains in 2-oxoglutarate dependent dioxygenases
Source: BMC Res Notes. 2012 Aug 4;5:410. doi: 10.1186/1756-0500-5-410 (PMC3475032; doi:10.1186/1756-0500-5-410)
Supplement: Additional file 4 — Table S4. Analysis of HMM profiles with highlighted active site residues. (PDF 29 kb) [file 1756-0500-5-410-S4.pdf]

Table S4: Details of HMM profiles used in this work

Profile	Structs.	Seqs	Sequence pattern of identical amino acids in alignment with highlighted active site residues[#]
**ALKB**	4	1	Nx{10,25}Hx{1}Dx{12,14}Sx{25,45}Gx{13,14}H
**ARGI**	2	1	Tx{7,8}ALAx{9,10}Dx{3}FLx{2}Ax{3}Ax{2}LPx{2}Lx{15,16}Lx{2}RGx{1}PVx{1,2}Dx{2}Lx{2}TPx{19}Lx{4}LGx{3}Gx{6}Gx{1}Vx{2}DVx{1}Px{2}Gx{1,2}Hx{1}Lx{1}Sx{2}Sx{3}Lx{2}HTEx{1}Ax{7}YVx{1}Lx{3}Rx{6,8}Tx{4}Vx{1,3}Lx{2}LDEx{1}Tx{3}Lx{9}Dx{15,30}Vx{2}Lx{1}Gx{4}Px{1}Lx{2}Dx{4}APx{1,3}Dx{5}Ax{3}Lx{4}Dx{7}Lx{2}GDx{4}DNx{1}Rx{2}Hx{1}Rx{2}Fx{1}PRx{1}DGx{1}DRWLx{1}Rx{2}Ix{2}D
**ASPA**	3	4	H
**CHLO**	2	1	Fx{9}Fx{3}Gx{1}IGPx{5}PEEMx{7}Rx{2}Lx{2}Rx{2}Ax{1}Yx{9,10}NYDRHLDx{1}Dx{1}Lx{2}Hx{2}Rx{1}EIx{2}RVx{1}Sx{2}Gx{4}CWRx{1}EFx{1}PKYx{1}Gx{1}EGTDWHQAx{1}TFAx{1}Ax{1}Gx{1}PQx{2}WPx{4,5}Fx{1}Gx{1}Ix{1}VWTx{1}FTx{5}NGCx{1}Qx{2}PGx{4,52}Ax{1}Px{3}Kx{1}Gx{3}IFx{1}Sx{2}MHx{1}Sx{1}Px{7,8}Rx{1}Gx{3}RYVPx{2}Vx{1}VYPx{13}Lx{3}Gx{1}Vx{3}Gx{6}NRx{7}G
**CLAS**	1	1	Mx{1}Sx{0,2}VDCTx{1}Yx{2}ELx{1}ALAx{2}LPx{2}PRADLx{2}FLDx{1}Ax{1}Tx{1}AAx{1}LPx{2}LAx{1}ALDTFNAx{1}GSEDGx{1}LLLRGLPVx{1,2}Dx{2}LPx{1}TPx{1}STPAPx{1}DRx{2}Lx{1}MEAMx{2}Lx{1}GRRLGLHTGYx{1}ELRSGTVYHDVYPSPGAHx{1}LSSETSETLLEFHTEMAYHx{1}LQPNYVMLACSRADHEx{2}Ax{1}TLVx{1}SVRKALPLLDEx{1}TRARLx{1}DRx{2}PCCVDVAFRGGVDDPGAIAx{1}VKPLYGDAx{1}DPFLGYDRELLAPEDPADKEAVAx{1}LSx{1}ALDx{1}VTx{2}Vx{1}Lx{1}PGDx{1}LIx{1}DNFRTTHARTPFSPRWDGKDRWLHRVYIRTDRNGx{1}LSGGERAGDx{3}Fx{1}PR
**COLY**	1[*]	13	Rx{130,172}W
**CP3H**	1[*]	9	Hx{27}Fx{65,73}Ex{17}Px{52,115}Px{2}Px{3,4}Fx{27,48}Sx{2}Hx{17}Sx{19,77}Vx{1}Ax{5}Rx{5}W

**CP4H**	3	6	----
**CYCL**	2	1	Sx{3,4}LLLRGx{7,27}Ex{41,48}Hx{4}Yx{4}Px{6}Cx{2}Ax{7,8}Tx{3}Sx{3}ALx{1}Lx{1}Dx{5,8}Rx{9,12}Ax{41,52}Ex{2}Ax{1}Lx{3}Lx{12,14}Dx{2}Ix{1}DNx{4}Hx{1}Rx{8,11}Rx{1}Lx{1}RVx{1}Ix{12}R
**DACS**	1	4	VPx{1}Fx{4}Lx{2}Gx{12,13}Gx{1}FYLx{3}Gx{14,15}DFFx{2}Gx{3}EKx{1}AVx{7}RRGx{3}LEx{1}ESTAx{2}Tx{1}TGx{1}Yx{1}DYSx{3}SMGx{3}Nx{1}FPx{3}FEx{2}Wx{2}YFx{3}Yx{1}Ax{4}ARx{1}VLx{3}Gx{14,17}Px{1}LRx{1}RYFPx{1}VPEx{1}Rx{1}AEx{2}Px{1}RMx{1}PHYDLSx{2}Tx{3}QTx{1}CANGFVSLQx{1}Ex{2}Gx{5}Px{4}Ax{2}Vx{1}CGAx{3}Lx{3}Gx{3}APx{1}Hx{1}Vx{2}Px{6}GSx{1}RTSSVFFLRPx{2}DFx{1}Fx{1}Vx{3}Rx{2}Gx{2}Vx{4}Ex{2}TFx{2}Wx{1}Gx{1}NY
**DSAT**	3	18	----
**ECTO**	1	3	Dx{1}YPx{1}Rx{8,11}Rx{1}Dx{1}Vx{8,11}Px{5}Lx{6}GFLx{8}DEVx{9}Lx{12}Ix{1}Ex{2}Sx{3}RSx{1}Fx{1}VHx{5}Fx{5}Dx{1}Rx{5}Rx{3}Gx{1}DVYVHQSRINx{1}KPGFx{4}Fx{1}WHSDFETWHx{1}EDGx{1}Px{1}Mx{3}Sx{1}SIx{1}Lx{2}Nx{3}NGx{1}LMx{2}PGSHx{2}Fx{2}Cx{1}Gx{1}TPx{4}Kx{1}SLx{2}Qx{2}Gx{1}Px{2}Ex{1}Lx{7}Gx{0,1}Ix{3}TGx{1}AGx{4}Fx{2}Nx{2}HGSx{2}Nx{2}Px{2}Rx{1}Nx{1}Fx{1}Vx{1}Nx{3}Nx{4}Px{6}RP
**FLAV**	1	17	Rx{3}Ex{13,19}Px{17,24}Ax{3}WGx{6}Hx{16}FFx{1}Lx{3}EKx{26,32}Wx{9}Px{8}WPx{2}Px{9}Yx{14}LSx{3}Gx{5}Lx{13,16}Nx{1}YPx{1}CPx{1}Px{1}Lx{1}LGx{3}HTDx{4}Tx{6}Vx{1}GLQx{6,8}Wx{14}GDx{5}SNGx{6}Hx{3}Vx{5}Rx{1}Sx{3}Fx{2}Px{7,8}P
**GBBH**	1*	3	Ax{5}Ax{6}Wx{1}Dx{3}Sx{5}WLRDNCx{1}Cx{1}DCx{10}Lx{23,24}Wx{3}Hx{1}Sx{5}WLx{7}Qx{5,7}Ex{3}Px{4}Wx{5}LPx{6}Vx{3}Dx{5}WLx{5}VGx{3}Lx{1}Gx{5}Gx{4}Lx{1}KRx{2}Fx{6}Gx{4}Vx{2}Kx{1}DAx{3}AYTx{3}Lx{2}HTDx{1}Px{5}PGx{5}C
**GIAC**	0	81	Gx{3}Hx{1}Dx{13}Gx{8,12}Wx{15,16}Dx{6}N

**HILY**	11	12	-----
**HP4H**	3	5	-----
**HYOS**	0	6	ATx{5}Sx{7,28}Fx{11}Vx{2}Gx{3,6}Px{1}Ix{1}Lx{7,13}Vx{2}Ix{2}Ax{4}Gx{1}FQx{2}NHGx{1}Px{3}Mx{9}Fx{18,26}Kx{2}LYx{13}Wx{1}DTx{13,17}Ex{1}Px{3}Rx{4}KYSx{3}Rx{1}Lx{9}Ex{1}LGLKx{14,15}Lx{2}YYPx{1}CPx{1}Px{2}Tx{1}Gx{3}Hx{1}Dx{4}Tx{1}Lx{1}Qx{3}Gx{2}Qx{6,7}WIx{1}Vx{2}IPx{1}Ax{1}Vx{1}Nx{4}Lx{4}Nx{1}KFx{4}HRVx{7}Rx{1}Sx{5}Gx{8,15}Px{1}KELx{4}NPPx{1}YKx{6}Fx{2}Ix{13}Px{3}N
**NUHY**	0	7	G
**OGFD**	0	2	Gx{6}Vx{3}Fx{21}Px{2}Hx{1}VIx{8,21}Lx{4}KELMx{3,4}Fx{1}EKx{2}Dx{1}Yx{3}Qx{1,7}Dx{1}Lx{1}Kx{8}LRx{4,8}Ex{1}FRSx{4}Ix{6,8}Sx{2}Dx{1}Sx{3}Yx{5}Lx{2}HDDx{4}Rx{1}Ix{2}ILYLVx{1}Px{0,3}Wx{4}GGx{1}Lx{1}Lx{6,7}Fx{7}Sx{2}Px{1}WNx{1}Lx{1}FFx{1}Vx{1}Px{1,2}SFHx{1}Vx{1}EVx{3,4}Kx{1}Rx{2}ISGWFHx{1}Px{4}Px{1,4}NYx{17}Lx{7}Yx{16}Sx{7}Lx{1}Px{4}Kx{9}Vx{31,33}SEx{2}Fx{9}Kx{3}Lx{4}Ex{4,12}DIx{9}Px{9}Sx{5}Sx{2}Qx{8,13}Sx{18}Lx{6}Ax{16}Px{13}Dx{2}LLx{6}Nx{3}LVYRDx{3}Lx{1}FVKx{10,19}WD
**PHYT**	2	3	Lx{3}Qx{7}Gx{1}LVx{51,82}Kx{36,38}Mx{1}Ix{1}Kx{1}Px{8,11}HQDx{7}Px{6,7}Wx{1}Ax{1}Ex{5}NGCLx{3}PGx{1}Hx{29,33}Vx{6}Gx{5}Hx{4}Hx{1}Sx{2}Nx{5}Rx{1}Ax{3}H
**SULF**	2	1	AAx{6}Px{3}Lx{47}Hx{3}Gx{13}Ix{7}Sx{11}Tx{18}Dx{7}Ax{49}Lx{9}Gx{2}Ix{1}Gx{2}Lx{3}Lx{16}Vx{3}Rx{1}Qx{17}Gx{4}Hx{36}WHTDVx{6}Px{10}Px{2}GGDTx{7}Ax{3}Lx{2}Px{7}Lx{3}Hx{19}Rx{13}HPx{1}VRx{1}HPx{6}Lx{5}Fx{3}Ix{7}Sx{2}Lx{3}Lx{12}Rx{2}Wx{6}IWDNRx{2}QHx{1}Ax{2}Dx{6}Rx{3}Rx{1}Tx{5}P
**TDLP**	1	1	Lx{6}Px{2}Gx{2}Ix{1}Gx{4,5}LSDx{5}Lx{3}Vx{4}VVx{2}Rx{1}Qx{3,6}Px{9}FGx{1}LHIHx{4}Hx{5}Ex{8,17}Dx{7}WHTDVx{5}PPx{8}Ex{1}Px{2}GGDTLx{4}Ix{1}Ax{3}LSx{1}Px{4}LSx{1}Lx{3}Hx{6,14}Ex{17}HPx{1}VRx{1}HPVx{2}Kx{2}Lx{1}VNx{2}Fx{3}IVx{5}ESEx{1}LLx{1}FLx{9,10}Qx{1}Rx{2}Wx{1}Px{4}IWDNRx{2}QHx{1}Ax{2}Dx{5,17}Rx{7}Lx{1}Dx{3}Y

**TFDA**	0	6	Px{6}Ax{6}Lx{2,3}Lx{19}Fx{2}Qx{5}Qx{1}Ix{1}Fx{3}FGx{8,23}Ix{16,25}Gx{3,6}WHx{1}DSx{13}Ax{2}VPx{2}GGx{1}Tx{1}Fx{2}Mx{2}Ax{3}Lx{14}Hx{8}Lx{18,27}PLVx{2}Hx{3}GRx{2}Lx{10,11}Gx{3}AEx{4}Lx{2}Lx{3}Ax{10}Wx{7}WDNx{1}Cx{2}Hx{10}R
**THYD**	0	2	Kx{3}Ex{1}Ax{20}Fx{12}Ax{1}Ex{3}Kx{3}Ex{11}Dx{4}Fx{5,18}Fx{4}Gx{4,7}Vx{1}IPx{3}Px{6}AAx{1}VLEx{1}Ax{5}LRx{4}GGx{2}Px{2}Gx{2}GYx{1}DYx{3}Px{3}KCRx{1}Tx{1}Fx{12}Px{8}Yx{4}Px{1}Hx{3}Qx{2}Ax{1}Px{6}Gx{2}FSTx{1}TVNx{2}FRTAx{1}HTDx{1}GDFx{2}Gx{1}GVLx{1}Vx{2}GEx{2}Gx{2}LAx{7}Fx{5}DVLLFDTx{1}Lx{1}Hx{1}NTEx{7,9}Wx{1}Rx{1}Sx{1}Vx{1}Yx{1}Rx{2}Lx{4}Cx{2}Ex{1}Rx{2}Lx{20}NGx{5}Px{4}Px{2}Lx{1,5}Px{3}AAx{6,12}GCx{2}AMx{3}Lx{0,32}Hx{5}Nx{3}Mx{2}Lx{8}DGLx{15,16}Nx{5}LGx{1,2}Fx{3}Gx{2}Lx{10,17}Dx{8}Gx{10}Rx{2,3}Lx{2}Vx{6}Qx{11}Wx{1}Nx{7,11}Fx{2}Lx{5}Qx{3}Gx{2}Gx{25}Nx{4}Lx{15}Yx{10}Mx{8}Rx{3}Lx{4}Ax{3}Ax{1}Ix{1}DSx{12,13}Qx{13}Ix{12}Ex{11}Gx{3}VLx{9}Rx{14,16}Ix{5}Ax{1}Rx{24}Nx{17}DFx{4}Hx{22}Rx{5}Vx{1}Vx{2}Lx{19,22}Fx{9}Rx{9}Cx{5}Dx{10}Kx{5,11}LNx{8}RE
**THYE**	0	2	Vx{5,7}Px{1}Ix{1}Fx{2}FLx{1}Gx{4}KLx{1}TAx{3}Lx{6}GFx{2}Lx{1}Nx{5}Dx{5,7}Fx{5}FFx{1}LPx{1}Ex{1}Kx{4}Wx{3}Ex{1}NRGYx{1}Ax{1}GREx{1}VTQx{1}TDPx{2}Ix{1}Kx{1}Rx{2}APDx{1}KEx{2}EIGx{5}Gx{0,1}Px{3}Px{1}Ex{6,10}FKx{2}MNx{1}FFx{5}LHx{1}Ex{1}MRAx{1}Ax{2}MGIx{4}FDx{2}Vx{6}LRLLx{1}YPx{2,4}Ex{2}KIx{2}Gx{2}RAGx{1}Hx{1}DYGSx{1}TLLx{1}QDx{2}GGLx{1}Vx{4}Gx{1}Fx{2}Ax{1}Px{3}TVVVNx{1}GDLLx{1}RWSNDx{2}KSTx{1}HRVVx{1}Px{7,9}Px{1}Rx{1}SIAx{1}Fx{1}NPx{5}Ix{1}Ax{1}PGTx{1,5}Ex{1}KYEx{6}YLVx{1}RLx{2}TY
**XANT**	0	3	Mx{3}Tx{6,10}Px{1}Gx{10,12}Dx{3}Lx{1}Dx{1}DFx{5}ALx{1}Tx{2}Vx{1}Vx{3}Qx{4}Px{2}QYx{2}Tx{1}Rx{1}DPx{4,6}YGHx{8,11}Sx{1}Lx{2}Dx{4}PHQPQVQx{2}Gx{1}Gx{5}EGx{5}Lx{1}HPx{1}Hx{1}TFHx{12,13}TRFYRWHIDx{1}ALYGx{2,5}PPx{2}TTLLx{3}VPx{4,5}Qx{3}Yx{1}Dx{1,2}Gx{9}Tx{1}Fx{1}SGx{6}LSx{6}Ax{4}Vx{1}YAPHPYx{3}Sx{1}Ax{5}GLx{3}Sx{3}Ex{5}LPx{3}Ex{5}LPMx{1}Wx{1}NPx{1}TGx{3}LQx{1}Hx{3}Vx{7}Gx{1}Vx{2,3}Lx{3}Rx{3}Yx{3}RPx{1}Ix{1}Px{2}VYAHx{1}Wx{2}GDLVx{1}FHNx{1}Gx{2}HSVx{1}Gx{1}Fx{3}Ex{1}RLx{2}QCNx{1}Ax{2,25}V

(#) Active site residues with side chains oriented towards HATM.
(*) PDB IDs in absence of HATM
HATM: atoms of Fe (II), 2-OG, and/or substrate
